# Protocol for mitochondrial DNA isolation from *Macrobrachium rosenbergii* and *Penaeus monodon* using differential centrifugation

**DOI:** 10.1016/j.xpro.2025.103852

**Published:** 2025-06-02

**Authors:** Dipta Chandra Pal, Muhammad Manjurul Karim

**Affiliations:** 1 Department of Microbiology, University of Dhaka, Dhaka 1000, Bangladesh

**Keywords:** Genomics, Molecular Biology, Sequence analysis, Sequencing

## Abstract

The mitochondrial genome (mitogenome) is a vital tool in molecular phylogenetics, evolutionary biology, and population genetics. Here, we present a protocol for extracting mitochondrial DNA from shrimp (*Macrobrachium rosenbergii*) and prawn (*Penaeus monodon*) species using a differential centrifugation approach without relying on commercially available kits. We describe steps for sample preparation, crude mitochondrial extraction, mitochondrial DNA isolation, and purity assessment. We then detail procedures for confirming the presence of the mitogenome and sequencing the mitochondrial genome.

For complete details on the use and execution of this protocol, please refer to Pal et al.^1^^,^^2^

## Highlights

• Instructions for enriching mitochondrial fractions using differential centrifugation
• Steps for extracting high-quality mitochondrial DNA
• Guidance on confirming mt-DNA using PCR amplification and gel electrophoresis
• Procedures for annotating mitochondrial genomes

## Abstract (editor highlights)

Publisher’s note: Undertaking any experimental protocol requires adherence to local institutional guidelines for laboratory safety and ethics.

## Graphical abstract

**Figure:**
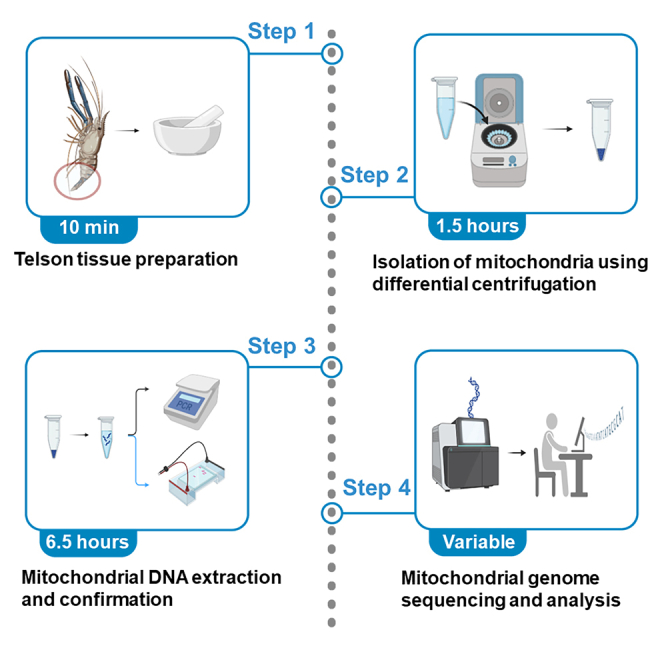

## Before you begin

The protocol described below outlines the specific steps used to isolate and sequence mitochondrial DNA from the telson tissue of *Macrobrachium rosenbergii* and *Penaeus monodon*. It was optimized for specimens collected from Bangladesh and has proven effective in yielding high-quality mitochondrial DNA suitable for genome sequencing and downstream bioinformatics analyses.^1^^,^^2^ The protocol can be adapted for other crustaceans and invertebrates with similar tissue structures by adjusting centrifugation speeds, buffer volumes, or incubation times. Researchers working with other taxa may modify the tissue homogenization and mitochondrial enrichment steps to accommodate differences in organismal physiology.

### Preparatory steps

1. To ensure the success of this protocol, prepare the workspace and materials in advance.
2. Maintain a sterile and contamination-free environment throughout the procedure to preserve the integrity of mitochondrial DNA.
3. Pre-cool all glassware, homogenizers, and centrifuge rotors in an ice bath prior to starting the experiment to prevent mitochondrial degradation.
4. Use freshly prepared Isolation Buffer and Lifton Buffer to ensure their effectiveness.
5. Confirm the availability of necessary equipment, such as a refrigerated centrifuge, heat block, spectrophotometer, and gel documentation system.
6. Prepare PCR reagents, primers (LCO1490 and HCO2198), and materials for agarose gel electrophoresis beforehand.
7. Verify the functionality of sequencing platforms and software tools such as FastQC, TrimGalore, SPAdes, MITOS, and OGDRAW for downstream applications.
8. Collect the telson tissue samples and process them promptly to avoid tissue degradation.

## Key resources table

**Table:** 

REAGENT or RESOURCE	SOURCE	IDENTIFIER
**Chemicals, peptides, and recombinant proteins**		

Tris-HCl	Sisco	79420
EDTA	Sisco	43272
EmeraldAmp GT PCR master mix	Takara Bio	RR901
Bovine serum albumin	Sigma-Aldrich	A-9418
Sodium chloride	Carl Roth	Art. No. 3957.1
Potassium chloride	Merck	104936
Di-sodium hydrogen phosphate	Merck	106586
Potassium dihydrogen phosphate	Merck	104873
D(−)-Mannitol	Merck	105982
Sucrose	FUJIFILM	196-00015
Sodium dodecyl sulfate	Sigma-Aldrich	436143
Phenol:chloroform:isoamyl alcohol (25:24:1, v/v)	Invitrogen	11518756
Ethanol	Merck	100983
Chloroform	Merck	102447
Isoamyl alcohol	Merck	100979
SeaKem LE agarose	Lonza	50002
Proteinase K	QIAGEN	51304

**Deposited data**		

*Macrobrachium rosenbergii* complete mitochondrial genome	This article/Pal et al.^1^	https://www.ncbi.nlm.nih.gov/nuccore/PQ213808.1
*Penaeus monodon* complete mitochondrial genome	This article/Pal et al.^2^	https://www.ncbi.nlm.nih.gov/nuccore/PQ876261.1

**Biological samples**		

*Macrobrachium rosenbergii*	This article/Pal et al.^1^	N/A
*Penaeus monodon*	This article/Pal et al.^2^	N/A

**Oligonucleotides**		

Primer for COI gene: forward (LCO1490): 5′-GGTCAACAAA TCATAAAGATATGG - 3’; reverse (HCO2198): 5′- TAAACTTCAGGGTGAC CAAAAAATCA – 3′	Costa et al.^3^	N/A

**Software and algorithms**		

FastQC v.0.12.112	Andrews (n.d.)^4^	https://github.com/s-andrews/FastQC
TrimGalore v.0.6.10	Krueger et al.^5^	https://github.com/FelixKrueger/TrimGalore
SPAdes v.3.15.5	Prjibelski et al.^6^	https://github.com/ablab/spades?tab=readme-ov-file
MITOS v.1.1.7	Bernt et al.^7^	https://gitlab.com/Bernt/MITOS/
OGDRAW	Greiner et al.^8^	https://chlorobox.mpimp-golm.mpg.de/OGDraw.html

**Other**		

Porcelain mortar with pestle	N/A	23-3505
High-speed refrigerated micro centrifuge	Tomy	MX-305
Starter 2100 pH bench pH meter	Ohaus	30057496
Microvolume spectrophotometer	Berthold	MX-305
Qubit 4 flurometer	Invitrogen	Q33238
Bioanalyzer 2100	Agilent	G2938A
Thermo mix HCM100-Pro	DLAB	5062103100
ProFlex PCR system	Applied Biosystems	4484073
Submarine electrophoresis system	Mupid One	MU2
Accessories (gel casting stand, gel trays, and combs)	Mupid One	ON-MS
Guardian 5000 hotplate stirrer	Ohaus	16640452
PR precision balance PR223/E	Ohaus	OH30430071
Corning Falcon centrifuge tubes	Sigma-Aldrich	352070
microDOC gel documentation hood with screen	Cleaver Scientific	CSL-MDOCUV254
UV transilluminator	Cleaver Scientific	CSLUVTS254
PCR cabinet	Esco	PCR-3A1
Class II biological safety cabinet	Esco	AC2-4D1
Laboratory water distiller	Wincom	WD-SS5
HP series laboratory freezer	Esco	HF2-400S-1
Microwave oven	Whirlpool	MW-20BS

## Materials and equipment

**Table:** Phosphate Buffered saline (PBS) pH 7.4

Reagent	Final concentration	Amount
NaCl	0.137 M	0.8 g
Na_2_HPO_4_	0.01 M	0.144 g
KCl	0.0027 M	0.02 g
KH_2_PO_4_	0.0018 M	0.025 g
ddH_2_O	N/A	up to 100 mL
Total	N/A	100 mL

Store at 4°C for up to 1 month.

**Table:** Isolation Buffer pH 7.4

Reagent	Final concentration	Amount
Mannitol	225 mM	4.1 g
Sucrose	75 mM	2.57 g
Bovine serum albumin	0.5%	0.5 g
EDTA	0.1 mM	2.922 mg
Tris–HCl	30 mM	0.336 g
ddH_2_O	N/A	Up to 100 mL
Total	N/A	100 mL

Prepare fresh for each experiment.

**Table:** Lifton Buffer pH 7.5^9^

Reagent	Final concentration	Amount
EDTA	100 mM	2.922 g
Tris-HCl	25 mM	0.303 g
SDS	1%	1 g
ddH_2_O	N/A	Up to 100 mL
Total	N/A	100 mL

Store at 4°C for up to 3 months.

## Step-by-step method details

### Preparing telson tissue for mitochondrial DNA extraction

**Timing: 10 min**

Here, we describe the initial steps required to prepare telson tissue for mitochondrial DNA extraction.

**Figure 1:**
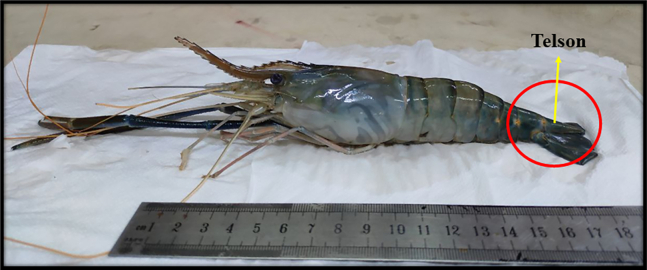
Sample (*Macrobrachium rosenbergii*) used for mitochondrial DNA extraction, highlighting the telson

***Note:*** The telson, located at the posterior end of the crustacean body (Figure 1), is carefully dissected from dead specimens to minimize contamination from surrounding tissues. The collected telson tissue is cleaned with sterile phosphate-buffered saline (PBS) to remove any residual debris or contaminants. This step ensures the isolation of high-quality tissue essential for efficient mitochondrial DNA extraction. Proper handling and preparation of the telson tissue are crucial to maintaining sample integrity and obtaining reproducible results in downstream analyses.

1. Separate the Telson.
  a. Carefully separate the telson part from the shrimp/prawn sample.
  b. Remove the shell and wash 3–4 times using ice-cold PBS.
  c. Cut the telson tissue into small pieces (2–3 mm) using sterilized scissors.
  d. Weigh the telson tissue using a precision balance (PR Precision Balance PR223/E).

### Isolation of crude mitochondria from shrimp/prawn telson tissue

**Timing: 1.5 h**

This step describes the isolation of crude mitochondria from the telson tissues of shrimp/prawn samples.

***Note:*** This protocol is an optimization of a previous protocol by Wieckowski et al.^10^ To ensure optimal results, it is essential to precool all the glassware, and the mortar and the pestle in an ice-bath before starting the procedure. To maintain sample integrity and prevent excessive degradation, the maximum recommended maceration time is 5 min. Prolonged maceration can lead to mitochondrial damage and reduced DNA yield.

**CRITICAL:** Avoid prolonged homogenization to prevent mitochondrial damage. Ensure all buffers and equipment are ice-cold.

**Figure 2:**
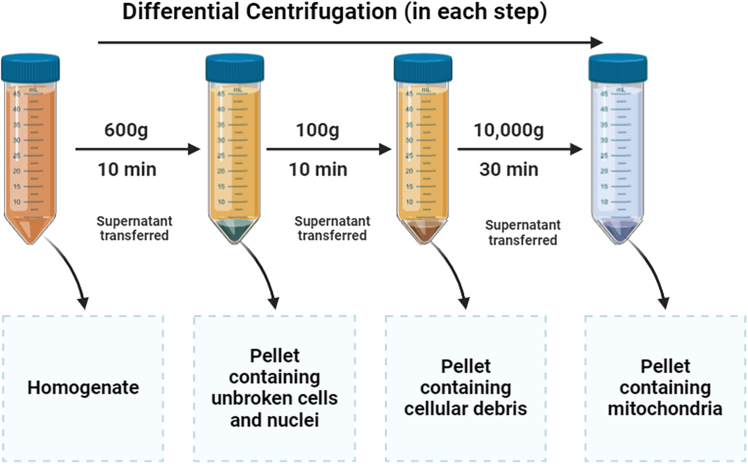
Mitochondria extraction using differential centrifugation technique

2. Homogenization of the telson tissue.
  a. Transfer the telson tissue pieces to a precooled mortar.
  b. Add ice-cold Isolation Buffer (pH 7.4) at a ratio of 4-6 mL per gram of telson tissue.
  c. Homogenize the tissue gently using a precooled pestle to minimize damage to mitochondrial structures.
3. Mitochondria isolation (Figure 2).
  a. Transfer the homogenate into a 50 mL Falcon Centrifuge Tube (approximately 10 mL) and centrifuge at 600 × *g* for 10 min at 4^o^C using a refrigerated centrifuge (Tomy MX-305 High Speed Refrigerated Micro Centrifuge).
  b. Carefully collect the supernatant into a fresh Falcon tube and discard the pellet containing unbroken cells and nuclei.
  c. Centrifuge at 100–200 × *g* for 5 min at 4^o^C using a refrigerated centrifuge.
  d. Transfer the resulting supernatant to another fresh Falcon tube and discard the pellet (if present).
  e. Centrifuge the final supernatant at 10,000 × *g* for 30 min at 4^o^C a refrigerated centrifuge.
  f. Discard the supernatant, which contains lysosomes and microsomes, and retain the pellet containing crude mitochondria.

***Note:*** This step can be performed with a minimum of 0.1 g of tissue, but in such cases, all centrifugation steps should be conducted using microcentrifuge (Eppendorf) tubes instead of 50 mL Falcon tubes. Additionally, this step has been successfully applied to frozen samples and other tissues, although with lower accuracy due to potential DNA degradation. To preserve DNA integrity, frozen samples should be stored at −26°C and thawed on ice just before use. Proper storage and minimal freeze–thaw cycles are essential for maintaining sample quality.

### Mitochondrial DNA extraction protocol

**Timing: 6.5 h**

This step outlines the extraction of high-quality mitochondrial DNA (mt-DNA) from mitochondrial pellet for downstream applications, including sequencing, PCR amplification, and genomic analysis. The goal is to isolate pure and intact mt-DNA while minimizing contamination from nuclear DNA and other cellular components.

***Note:*** After extraction, confirmation of mt-DNA is achieved via gel electrophoresis and/or PCR using mitochondrial-specific markers, ensuring the integrity and specificity of the extracted DNA. This step is critical for accurate and reproducible results, as contamination or degraded DNA can compromise data quality.

4. Mitochondrial DNA extraction.
  a. Add 400 μL Lifton Buffer and 15 μL proteinase K (20 mg/mL) to the mitochondrial pellet.
  b. Incubate the sample at 60^o^C for 3 h (until the solution becomes clear) in a thermomixer.
  c. Add an equal volume of PCI (Phenol: Chloroform: Isoamyl Alcohol, 25:24:1), hereafter 400 μL to the lysate.
  d. Centrifuge at 7,200 × *g* for 10 min at 4^o^C.
  e. Carefully collect the aqueous layer (approximate 300 μL) in the supernatant.
  f. Add 300 μL (Chloroform: Isoamyl Alcohol, 24:1) to the collected supernatant.
  g. Centrifuge at 7,200 × *g* for 10 min at 4^o^C and collect the top aqueous layer (approximately 100 μL).
  h. Add Ice-cold absolute ethanol 700 μL and mix gently.
  i. Incubate the tube at −20^o^C for 10 min (Incubation time can be increased if the sample size is minimal).
  j. Centrifuge at 18,000 × *g* for 10 min at 4^o^C and discard the supernatant.
  k. Wash the pellet with 70% ethanol (500–600 μL) and centrifuge at 18,000 × *g* for 10 min at 4^o^C (repeat the step if necessary).
  l. Discard supernatant and allow the pellet to air dry at 25°C for 10 min.
  m. Dissolve the pellet in nuclease-free water (30/40 μL).
  n. Evaluate the quality of the extracted DNA using a spectrophotometer (e.g. Colibri-Model LB 915 microvolume spectrophotometer, Berthold Technologies GmbH & Co. KG, Germany).

***Note:*** Measure DNA concentration using a spectrophotometer and check integrity via gel electrophoresis. A_260_/A_280_ ratio should be ∼1.8–2.0 for pure DNA.

**Figure 3:**
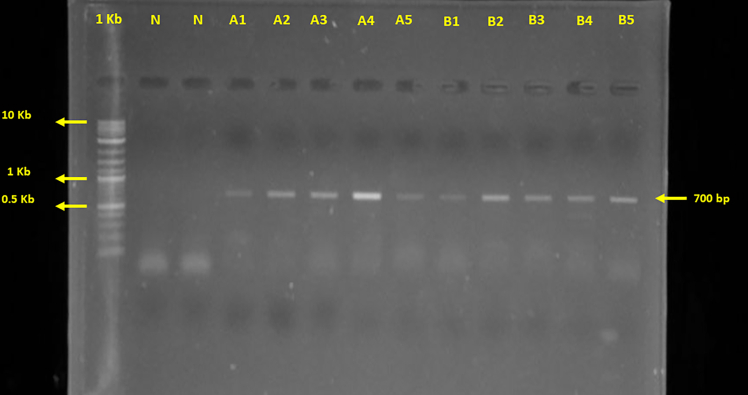
PCR product amplified from the COI gene of *Macrobrachium rosenbergii* and *Penaeus monodon* electrophoresed on a 1% agarose gel Here, N = Negative, A = *P. monodon,* B *= M. rosenbergii.*

5. Confirm the presence of mitochondrial DNA.
  a. Run mitochondrial COI gene specific PCR using the primer LCO1490 (Forward) and HCO2198 (Reverse) on ProFlex PCR System (Tables 1 and 2).
  b. Load 20% PCR product to 1% agarose gel and run gel electrophoresis at 100 V for 35 min.
  c. Check the gel in a gel documentation system (Figure 3).

***Note:*** PCR reaction mix and PCR cycling conditions are listed in Tables 1 and 2, respectively.

**Table 1:** PCR reaction master mix

Reagent	Amount (μL)
DNA template	Volume for 20 ng
EmeraldAmp GT PCR Master Mix	12.5
Primer Forward (10 pmol/μL)	2.5
Primer Reverse (10 pmol/μL)	2.5
ddH_2_O	Volume to make the 25 μL total
Total	25

**Table 2:** PCR cycling conditions

Steps	Temperature	Time	Cycles
Initial Denaturation	94 °C	4 min	1
Denaturation	94 °C	30 s	30 cycles
Annealing	50 °C	45 s	
Extension	72 °C	1 min	
Final extension	72 °C	6 min	1
Hold	4 °C	forever

### Mitochondrial genome sequencing and analysis

**Timing: Variable**

This step involves sequencing and analyzing the complete mitochondrial genome to uncover the genetic architecture and features of the organism.

***Note:*** Extracted mitochondrial DNA is subjected to high-throughput sequencing using platforms such as Illumina, ensuring sufficient coverage and accuracy. This step is critical for generating high-quality genomic data and provides insights into mitochondrial features. Proper computational analysis ensures accurate annotation and reliable results for further research.

**Figure 4:**
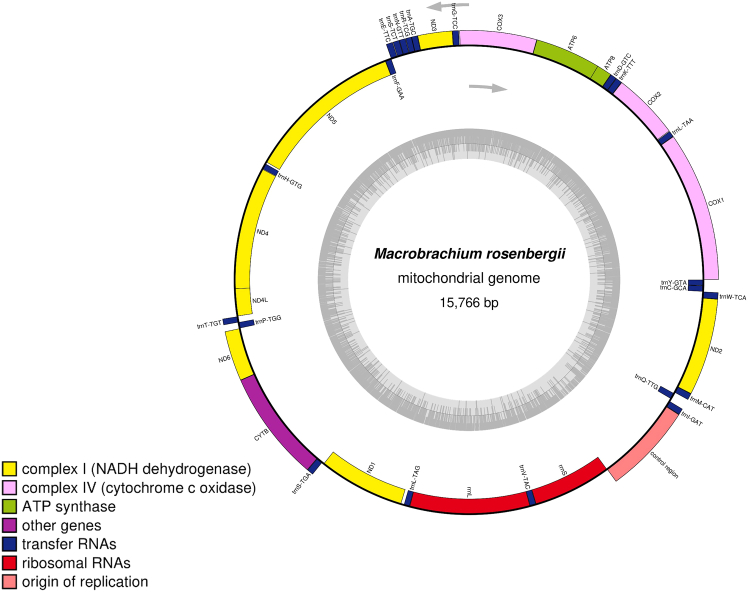
The complete mitochondrial genome map of *Macrobrachium rosenbergii* The innermost ring illustrates the GC content, shown in ash gray, with transcription directions indicated by arrows. The outermost ring displays the genes, with those transcribed clockwise placed on the inside of the circle and those transcribed counterclockwise on the outside. The color coding corresponds to different gene groups, as indicated in the key located in the bottom left corner. The annotated map includes 13 protein-coding genes, 2 ribosomal RNA genes (rrnS for 12S ribosomal RNA and rrnL for 16S ribosomal RNA), 22 transfer RNA genes, and the putative control region.^1^

**Figure 5:**
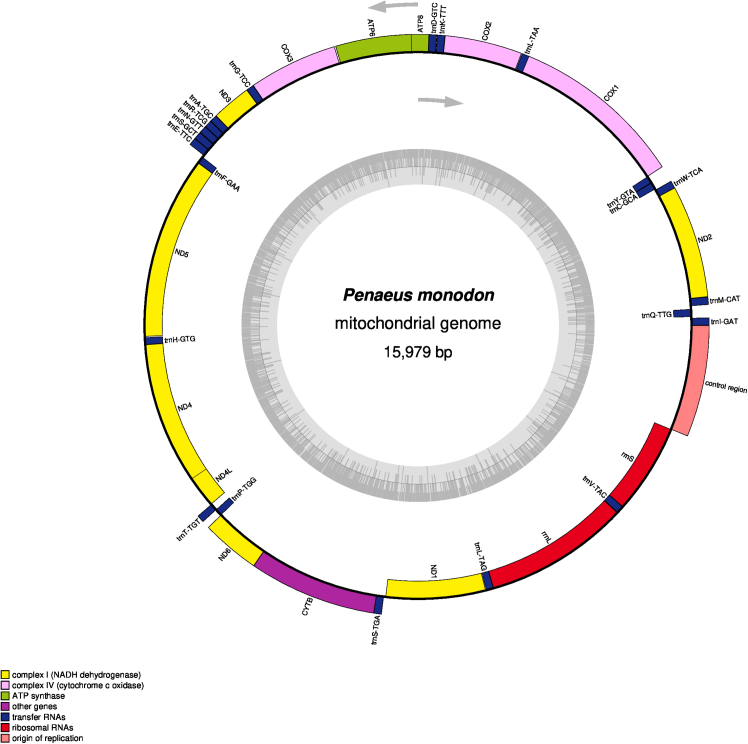
The mitochondrial genome map of *Penaeus monodon* It shows GC content in the innermost ring (ash grey), transcription directions as arrows, with clockwise-transcribed genes on the inner side and counterclockwise-transcribed genes on the outer side, color-coded gene groups (key in the bottom left corner) including 13 protein-coding genes (PCGs), 2 ribosomal RNA genes (rrnS for 12S and rrnL for 16S rRNA), 22 transfer RNA (tRNA) genes, and the putative control region.^2^

6. Perform sequencing.
  a. Conduct whole mitochondrial genome sequencing on the Illumina NextSeq 550 platform using a paired-end (2 × 151 bp) sequencing strategy to ensure high coverage and accuracy.
  b. Use the Illumina DNA Prep kit (Illumina, San Diego, CA, USA) for library preparation according to the manufacturer’s standard protocol, ensuring the fragmentation and adapter ligation steps are optimized for mitochondrial DNA sequencing.
  c. Perform quantification and quality control (QC) of the prepared libraries using Qubit (Invitrogen, USA) and Bioanalyzer (Agilent, USA) before sequencing.
7. Analysis of sequencing data.
  a. Assess the raw sequencing data quality using FastQC v.0.12.112, and filter out low-quality reads (Q < 20) using TrimGalore v.0.6.10 with default settings.
  b. Conduct *de novo* genome assembly of the trimmed reads using SPAdes genome assembler v.3.15.5.
  c. Perform gene annotation using MITOS v.1.1.7 with the Invertebrate Mitochondrial Code (transl_table=5) for accurate translation of mitochondrial genes.
  d. Verify the gene annotations using NCBI nucleotide BLAST against mitochondrial reference genomes.
  e. Visualize circular mitomap using OrganellarGenomeDRAW (OGDRAW) (Figures 4 and 5).

***Note:*** Validate mitochondrial contigs using BLAST against reference genomes. Use MITOS for automated gene annotation and manually curate key regions by NCBI nucleotide BLAST results against mitochondrial reference genomes.

## Expected outcomes

The expected outcome of this protocol is the successful isolation of high-quality mitochondrial DNA from shrimp or prawn telson tissues, suitable for downstream applications such as PCR amplification, sequencing, and mitogenomic analyses. The mitochondrial DNA should appear clean and free from significant contamination, with spectrophotometric analysis yielding a high purity ratio (A_260_/A_280_ between 1.8 and 2.0). PCR amplification using COI gene-specific primers is anticipated to produce a clear, distinct band of the expected size (∼700 bp) on a 1% agarose gel, confirming the presence of mitochondrial DNA (Figure 3). Sequencing on the Illumina NextSeq 550 platform should generate high-quality reads (Q ≥ 20) that allow for accurate de novo assembly of the mitochondrial genome. Annotation and visualization of the genome using tools like MITOS and OGDRAW are expected to produce a complete, annotated mitogenome with clearly defined genes and a circular mitomap (Figures 4 and 5). This outcome will provide valuable genetic insights for molecular phylogeny, evolutionary, biogeographic relationships, aquaculture management, and conservation studies of the target species.

## Limitations

The protocol for mitochondrial DNA isolation and sequencing from shrimp/prawn telson tissues has certain limitations that should be considered. The yield and purity of mitochondria are highly dependent on the quality and freshness of the tissues; degraded or poorly preserved samples may result in low yield or contamination. The isolation process, though optimized, may inadvertently include nuclear, lysosomal, or microsomal components, affecting downstream analyses. Additionally, the success of mitochondria isolation relies on the quality of reagents, particularly during differential centrifugation using the Isolation Buffer, where pH variation, contamination, or improper handling can reduce the mitochondrial yield. Finally, the specificity of primers for mitochondrial COI gene amplification can vary due to inter-species sequence differences, potentially leading to unsuccessful PCR amplification or misidentification. These factors should be carefully addressed to ensure the protocol’s reliability and reproducibility.

## Troubleshooting

### Problem 1

Tissue becomes mushy or difficult to homogenize (related to Step 1b).

### Potential solution

Ensure tissue is always kept on ice and precool the glassware and mortar with pestle in an ice-bath before starting the homogenization. Avoid over-washing with PBS.

### Problem 2

Poor mitochondrial yield (related to Step 2).

### Potential solution

Check the integrity of the tissue and ensure proper homogenization. Increase the number of telson tissue pieces if needed.

### Problem 3

Contamination with unbroken cells or nuclei (related to Step 3b and 3d).

### Potential solution

Ensure careful transfer of supernatant after each centrifugation step. Increase centrifugation time slightly (by 10%) if needed.

### Problem 4

Low mt-DNA yield or degraded mt-DNA (related to Step 4a and 4h).

### Potential solution

Use fresh reagents and ensure ethanol precipitation is performed properly. Minimize exposure of DNA to room temperature (> 25°C).

### Problem 5

No band observed on the gel (related to Step 5a).

### Potential solution

Verify the primer sequences and ensure proper PCR conditions (e.g., annealing temperature).

## Resource availability

### Lead contact

Further information and requests for resources and reagents should be directed to and will be fulfilled by the lead contact, Muhammad Manjurul Karim (manjur@du.ac.bd).

### Technical contact

Technical questions on executing this protocol should be directed to and will be answered by the technical contact, Dipta Chandra Pal (dipta.chandra1234@gmail.com).

### Materials availability

This study did not generate new unique reagents.

### Data and code availability

The published article includes all datasets and code generated or analyzed during this study.

## Acknowledgments

The authors gratefully acknowledge the financial support provided by the Biotechnology Research Centre, University of Dhaka (dated March 20, 2024).

## Author contributions

D.C.P.: data curation, formal analysis, methodology, software, and writing – original draft. M.M.K.: conceptualization, funding acquisition, investigation, project administration, resources, supervision, validation, and writing – review and editing.

## Declaration of interests

The authors declare no competing interests.

## References

[1] Pal D.C., Hasan M.M., Khan S.N., Karim M.M. (2025). Complete mitochondrial genome sequence of giant freshwater prawn, *Macrobrachium rosenbergii* of Bangladesh. Microbiol. Resour. Announc..

[2] Pal D.C., Khan S.N., Karim M.M. (2025). Complete mitochondrial genome sequence of giant tiger prawn, *Penaeus monodon*, of Bangladesh. Microbiol. Resour. Announc..

[3] Costa F.O., DeWaard J.R., Boutillier J., Ratnasingham S., Dooh R.T., Hajibabaei M., Hebert P.D. (2007). Biological identifications through DNA barcodes: The case of the Crustacea. Can. J. Fish. Aquat. Sci..

[4] Andrews S. (2020). https://www.bioinformatics.babraham.ac.uk/projects/fastqc/.

[5] Krueger F., James F., Ewels P., Afyounian E., Weinstein M., Schuster-Boeckler B., Hulselmans G., Sclamons (2023). TrimGalore: v0.6.10 - add default decompression path (0.6.10). Zenodo.

[6] Bankevich A., Nurk S., Antipov D., Gurevich A.A., Dvorkin M., Kulikov A.S., Lesin V.M., Nikolenko S.I., Pham S., Prjibelski A.D. (2012). SPAdes: A new genome assembly algorithm and its applications to single-cell sequencing. J. Comput. Biol..

[7] Bernt M., Donath A., Jühling F., Externbrink F., Florentz C., Fritzsch G., Pütz J., Middendorf M., Stadler P.F. (2013). MITOS: Improved de novo metazoan mitochondrial genome annotation. Mol. Phylogenet. Evol..

[8] Greiner S., Lehwark P., Bock R. (2019). OrganellarGenomeDRAW (OGDRAW) version 1.3.1: Expanded toolkit for the graphical visualization of organellar genomes. Nucleic Acids Res..

[9] Nishiguchi M.K., Doukakis P., Egan M., Kizirian D., Phillips A., Prendini L., Rosenbaum H.C., Torres E., Wyner Y., DeSalle R. (2002). Techniques in Molecular Systematics and Evolution.

[10] Wieckowski M.R., Giorgi C., Lebiedzinska M., Duszynski J., Pinton P. (2009). Isolation of mitochondria-associated membranes and mitochondria from animal tissues and cells. Nat. Protoc..

